# The combined impact of time in range and HOMA-IR on neonatal outcomes in gestational diabetes mellitus: A retrospective cohort study

**DOI:** 10.1097/MD.0000000000048074

**Published:** 2026-03-13

**Authors:** Mali Chen, Jun Sun, Jing Yang, Jianfen Wang, Chengzhao Shu, Lili Zhang, Xiaoli Chai, Yan Dong

**Affiliations:** a Department of Obstetrics, Gansu Provincial Maternity and Child-Care Hospital (Gansu Provincial Central Hospital), Lanzhou, Gansu, China; b The First Clinical Medical College, Gansu University of Chinese Medicine, Lanzhou, Gansu, China; c Department of Obstetrics, Pingliang Maternity and Child-Care Hospital, Gansu, China.

**Keywords:** gestational diabetes mellitus, HOMA-IR, insulin resistance, neonatal outcomes, time in range

## Abstract

Time in range (TIR) derived from continuous glucose monitoring and the Homeostatic Model Assessment of Insulin Resistance (HOMA-IR) are independent predictors of maternal and neonatal outcomes in gestational diabetes mellitus (GDM). However, their combined effect on neonatal outcomes remains unclear. This study aimed to investigate the joint influence of TIR and HOMA-IR on neonatal outcomes in GDM patients. In this retrospective cohort study, 166 women with GDM were categorized into 4 groups based on TIR levels (cutoff: 90%) and HOMA-IR (cutoff: cohort median): Group A (high-TIR/low-IR), Group B (high-TIR/high-IR), Group C (low-TIR/low-IR), and Group D (low-TIR/high-IR). HOMA-IR was calculated at “24 to 28 weeks’ gestation” gestation using fasting plasma glucose and insulin levels obtained during the oral glucose tolerance test. Neonatal complications, including hypoglycemia, macrosomia, and hyperbilirubinemia, were compared across groups. Compared with Group A (high-TIR/low-IR), Group D (low-TIR/high-IR) demonstrated the highest risk for adverse neonatal outcomes, with significantly higher incidence of neonatal hypoglycemia (OR: 4.52, 95% CI: 1.89–10.78) and macrosomia (OR: 3.45, 95% CI: 1.45–8.19), along with higher mean neonatal bilirubin levels (10.97 ± 1.72 mg/dL vs 8.99 ± 2.09 mg/dL, *P* < .001). Poor glycemic control (low TIR) combined with significant insulin resistance (high HOMA-IR) identifies a subgroup of GDM patients at the highest risk for adverse neonatal outcomes. The joint assessment of TIR and HOMA-IR may facilitate precise risk stratification and personalized management in clinical practice.

## 1. Introduction

Gestational diabetes mellitus (GDM) is one of the most common medical complications during pregnancy, with a rising global prevalence that poses significant health challenges for both mothers and their offspring.^[1]^ While maternal hyperglycemia is the hallmark of GDM, the underlying pathophysiological mechanisms and subsequent clinical manifestations are heterogeneous, primarily revolving around impaired pancreatic β-cell function and escalating insulin resistance (IR) as pregnancy progresses.^[2]^ This metabolic dysregulation creates a intrauterine environment exposed to excess glucose and other nutrients, which is strongly associated with a spectrum of adverse neonatal outcomes, including macrosomia, neonatal hypoglycemia, and hyperbilirubinemia.^[3,4]^

The cornerstone of GDM management is glycemic control. Traditionally, this has been monitored using hemoglobin A1c and self-monitored blood glucose.^[5]^ However, these metrics offer limited insights into daily glucose fluctuations. The advent of continuous glucose monitoring (CGM) has revolutionized this landscape by providing a comprehensive picture of glycemic variability. Among CGM-derived metrics, time in range (TIR), defined as the percentage of time spent within the target glucose range (3.5–7.8 mmol/L), has emerged as a robust and intuitive indicator of glycemic status and a strong predictor of diabetic complications.^[6,7]^ In GDM, a lower TIR has been independently linked to an increased risk of LGA infants and other morbidities.^[8]^

Concurrently, the Homeostatic Model Assessment of Insulin Resistance (HOMA-IR) serves as a well-validated and practical tool for quantifying the degree of IR.^[9]^ In GDM, a higher HOMA-IR value is not merely a pathophysiological marker but also a significant clinical risk factor, associated with a greater need for pharmacotherapy and an elevated risk of delivering macrosomic infants.^[10,11]^ This suggests that the inherent metabolic burden of IR contributes independently to adverse outcomes.

Despite their established individual predictive values, a critical gap exists in our understanding of how these 2 key parameters – dynamic glycemic control (TIR) and static metabolic burden (HOMA-IR) – interact. It remains unclear whether optimal glycemic control (high TIR) can mitigate the risks imposed by high underlying IR, or if the combination of poor control and high resistance creates a particularly detrimental clinical scenario.^[12]^

Therefore, this study aimed to investigate the combined impact of TIR and HOMA-IR on neonatal outcomes in GDM. We hypothesized that the combination of high IR (high HOMA-IR) and suboptimal glycemic control (low TIR) would identify a subgroup of GDM patients at the greatest risk for adverse neonatal outcomes, and that maintaining a high TIR could mitigate the risks associated with IR.

## 2. Methods

### 2.1. Study population and design

This retrospective cohort study was conducted at Department of Obstetrics, Gansu Provincial Maternal and Child Health Hospital and approved by the Institutional Review Board (IRB No: (2022)GSFY-51). The requirement for informed consent was waived due to the retrospective nature of the study.

#### 2.1.1. Inclusion and exclusion criteria

Pregnant women diagnosed with GDM according to the International Association of Diabetes and Pregnancy Study Groups criteria who underwent CGM between 28 and 32 weeks of gestation were initially screened for eligibility.^[13]^

The exclusion criteria were as follows:

1. preexisting type 1 or type 2 diabetes.
2. Multiple gestations.
3. Major fetal congenital anomalies.
4. Use of insulin or other glucose-lowering medications prior to or during the CGM period. This criterion was applied to isolate the independent effects of TIR and HOMA-IR without the confounding influence of pharmacological glucose-lowering therapy.
5. Incomplete clinical or laboratory data.

#### 2.1.2. Data collection and definitions

Demographic and clinical data, including maternal age, gestational age, parity, pre-pregnancy weight, and height, were extracted from electronic medical records. Pre-pregnancy body mass index (BMI) was calculated as weight (kg) divided by height squared (m^2^).

Fasting plasma glucose and insulin levels used for the HOMA-IR calculation were obtained during the diagnostic 75-gram oral glucose tolerance test at 24 to 28 weeks of gestation. HOMA-IR was calculated using the formula: [Fasting insulin (μIU/mL) × Fasting glucose (mmol/L)]/22.5.

### 2.2. Grouping criteria

#### 2.2.1. Definition of exposure groups

Participants were categorized into 4 groups based on 2 key parameters: Time in range (TIR): Defined as the percentage of time spent within the glucose target range of 3.5 to 7.8 mmol/L during the CGM period. A cutoff of 90% was used, consistent with current consensus targets for pregnancy^[14]^; and HOMA-IR: A cutoff value of 2.0 was selected to define high IR. This cutoff was chosen based on its association with adverse metabolic profiles in our specific GDM population and for exploratory analysis, as established, universally accepted HOMA-IR cutoffs for pregnancy are lacking. We acknowledge this as a potential limitation in the Discussion.

The 4 exposure groups were: Group A (high-TIR/low-IR): TIR ≥ 90% and HOMA-IR < 2.0; Group B (high-TIR/high-IR): TIR ≥ 90% and HOMA-IR ≥ 2.0; Group C (low-TIR/low-IR): TIR < 90% and HOMA-IR < 2.0; and Group D (low-TIR/high-IR): TIR < 90% and HOMA-IR ≥ 2.0.

### 2.3. Outcome measures

#### 2.3.1. Maternal outcomes

Maternal outcomes included the incidence of gestational hypertension, preeclampsia, and mode of delivery. “Assisted delivery” was defined as operative vaginal delivery, including forceps or vacuum extraction. Cesarean delivery was analyzed separately.

#### 2.3.2. Neonatal outcomes

Neonatal outcomes were assessed at birth and during the immediate postnatal period. The primary outcomes of interest were:

1. Neonatal hypoglycemia: Defined as a measured blood glucose level < 2.2 mmol/L within 24 hours of birth.
2. Macrosomia: Defined as a birth weight > 4000 grams.
3. Neonatal hyperbilirubinemia: Defined as requiring phototherapy based on pediatric assessment and standard nomograms.

Secondary outcomes included birth weight, gestational age at delivery, and Apgar scores at 1 and 5 minutes.

### 2.4. Statistical analysis

Continuous variables were presented as mean ± standard deviation or median (interquartile range) based on their distribution and were compared using Student *t* test, ANOVA, or the Mann–Whitney *U*/Kruskal–Wallis test as appropriate. Categorical variables were presented as numbers (percentages) and compared using the χ^2^ test or Fisher exact test.

To assess the independent and combined effects of TIR and HOMA-IR on neonatal outcomes, multivariate logistic regression models were constructed. The models initially included the TIR group, the IR group, and their interaction term. Potential confounders considered for adjustment in the models included maternal age, pre-pregnancy BMI, gestational weight gain, and parity. The final model was determined using a stepwise selection process with a significance level of 0.1 for entry and 0.05 for retention. The results were expressed as adjusted odds ratios with 95% confidence intervals.

A two-tailed *P*-value < .05 was considered statistically significant. All statistical analyses were performed using SPSS Statistics version 26.0 (IBM Corp., Armonk).

## 3. Results

### 3.1. Baseline maternal characteristics

A total of 166 women with GDM were included in the final analysis and categorized into 4 groups based on their TIR and HOMA-IR levels. The baseline clinical and biochemical characteristics of the participants are summarized in Table 1.

**Table 1:** Baseline maternal characteristics stratified by TIR and HOMA-IR groups.

Characteristic	Group A (n = 41)	Group B (n = 33)	Group C (n = 45)	Group D (n = 47)	*P*-value
Maternal age (yr)	30.2 ± 3.1	31.5 ± 3.8	31.0 ± 3.5	32.1 ± 3.9	.089
Gestational age at CGM (wk)	30.1 ± 1.2	29.8 ± 1.4	29.9 ± 1.3	30.2 ± 1.1	.456
Pre-pregnancy BMI (kg/m^2^)	21.8 ± 2.5	24.1 ± 3.2	23.5 ± 2.9	26.3 ± 3.8	<.001
Height (m)	1.61 ± 0.05	1.59 ± 0.04	1.62 ± 0.05	1.60 ± 0.06	.067
Diabetes family history, n (%)	8 (19.5)	7 (21.2)	9 (20.0)	10 (21.3)	.997
Fasting glucose (mmol/L)	4.5 ± 0.4	4.8 ± 0.5	4.7 ± 0.5	5.1 ± 0.6	<.001
HbA1c (%)	5.57 ± 0.32	5.59 ± 0.35	5.61 ± 0.33	5.65 ± 0.38	.223

BMI = body mass index, CGM = continuous glucose monitoring, HbA1c = hemoglobin A1c, HOMA-IR = Homeostatic Model Assessment of Insulin Resistance, TIR = time in range.

Maternal age, gestational age at CGM, and height were comparable across all groups. However, pre-pregnancy BMI and fasting glucose levels showed statistically significant differences (*P* < .001), with Group D (low-TIR/high-IR) exhibiting the highest values. While the mean hemoglobin A1c levels were very similar across groups (ranging from 5.57–5.65%), the overall *P*-value of .223 indicated no statistically significant difference.

### 3.2. Maternal pregnancy outcomes

Maternal pregnancy outcomes are detailed in Table 2. The incidence of gestational hypertension and preeclampsia did not differ significantly among the groups. Regarding delivery mode, while the cesarean delivery rate was higher in Group D (51.79%) compared to Group A (38.25%), this difference did not reach statistical significance (*P* = .131). The rates of assisted delivery (defined as operative vaginal delivery with forceps or vacuum) and preterm birth were also similar across the groups.

**Table 2:** Maternal pregnancy outcomes.

Outcome	Group A (n = 41)	Group B (n = 33)	Group C (n = 45)	Group D (n = 47)	*P*-value
Gestational hypertension, n (%)	5 (12.20)	4 (12.12)	6 (13.33)	7 (14.89)	.974
Preeclampsia, n (%)	2 (4.88)	1 (3.03)	3 (6.67)	3 (6.38)	.892
Cesarean delivery, n (%)	16 (38.25)	14 (42.42)	19 (42.22)	24 (51.79)	.131
Assisted delivery, n (%)	3 (7.32)	2 (6.06)	4 (8.89)	4 (8.51)	.964
Preterm birth (<37 wk), n (%)	2 (4.88)	2 (6.06)	3 (6.67)	4 (8.51)	.904

### 3.3. Neonatal outcomes

Neonatal outcomes are presented in Table 3. Group D (low-TIR/high-IR) had the highest incidence of adverse neonatal outcomes.

**Table 3:** Neonatal outcomes.

Outcome	Group A (n = 41)	Group B (n = 33)	Group C (n = 45)	Group D (n = 47)	*P*-value
Birth weight (g)	3250 ± 350	3320 ± 380	3410 ± 410	3680 ± 450	<.001
Macrosomia, n (%)	3 (7.32)	4 (12.12)	5 (11.11)	11 (23.40)	.048
Neonatal hypoglycemia, n (%)	4 (9.76)	5 (15.15)	6 (13.33)	13 (27.66)	.032
Neonatal bilirubin level (mg/dL), mean ± SD	8.99 ± 2.09	9.45 ± 1.95	9.88 ± 1.81	10.9 ± 1.72	<.001
5-min Apgar score < 7, n (%)	1 (2.44)	1 (3.03)	1 (2.22)	2 (4.26)	.921

The incidence of macrosomia and neonatal hypoglycemia was significantly higher in Group D compared to other groups (*P* < .05). Furthermore, mean neonatal bilirubin levels were significantly higher in Group D (10.97 ± 1.72 mg/dL) compared to Group A (8.99 ± 2.09 mg/dL, *P* < .001).

### 3.4. Other pregnancy complications

Other maternal and neonatal complications are listed in Table 4. The incidence of amniotic fluid abnormalities and neonatal intensive care unit admission did not differ significantly. A critical review of the data revealed errors in the initial presentation of amniotic fluid and postpartum hemorrhage data, which have been corrected in the table below.

**Table 4:** Other pregnancy complications.

Complication	Group A (n = 41)	Group B (n = 33)	Group C (n = 45)	Group D (n = 47)	*P*-value
Amniotic fluid-normal, n (%)	27 (65.85)	22 (66.67)	30 (66.67)	29 (61.70)	.956
Amniotic fluid-MSAF grade I, n (%)	3 (7.32)	2 (6.06)	3 (6.67)	4 (8.51)	.968
Amniotic fluid-MSAF grade II/III, n (%)	1 (2.44)	1 (3.03)	2 (4.44)	2 (4.26)	.942
NICU admission, n (%)	2 (4.88)	2 (6.06)	3 (6.67)	5 (10.64)	.701
Postpartum hemorrhage, n (%)	3 (7.32)	4 (12.12)	4 (8.89)	5 (10.64)	.892

MSAF = meconium stained amniotic fluid, NICU = neonatal intensive care unit.

## 4. Discussion

The principal finding of this study is that among women with GDM, the combination of suboptimal glycemic control (low TIR) and significant IR (high HOMA-IR) identifies a subgroup at the highest risk for adverse neonatal outcomes. Specifically, patients exhibiting both low TIR and high HOMA-IR (Group D) demonstrated a significantly greater incidence of neonatal hypoglycemia, macrosomia, and hyperbilirubinemia compared to all other groups.

### 4.1. Interpretation of key findings

Our results are consistent with, and significantly extend, the existing literature. Previous studies have independently established the prognostic value of both TIR and HOMA-IR for pregnancy outcomes. The novelty of our study lies in the concurrent evaluation of these 2 key parameters. We observed that even among patients with high TIR (Group B), the presence of high HOMA-IR was associated with a trend towards increased risk, underscoring the intrinsic metabolic burden imposed by IR, independent of immediate glycemic control. Conversely, among patients with low TIR (Groups C and D), those with concomitant high HOMA-IR (Group D) faced the most pronounced risk. This pattern suggests a significant combined effect of low TIR and high HOMA-IR on adverse neonatal outcomes. The non-significant statistical interaction term indicates that this combined effect is best described as additive rather than synergistic. From a pathophysiological perspective, a state of high IR may exacerbate fetal overgrowth and metabolic disturbances in a hyperglycemic environment by promoting maternal lipolysis and increasing the transfer of free fatty acids to the fetus.^[15]^

### 4.2. Comparison with previous studies

In contrast to the findings of Raj et al^[16]^ in type 2 diabetes, our study focuses specifically on GDM. While we adopted their conceptual framework regarding the importance of TIR, we found that the prognostic significance of glycemic variability is more complex in the context of the inherent IR of GDM. Our results emphasize that focusing solely on glucose levels in GDM management may be insufficient; a concurrent assessment of the patient’s IR status is necessary for more precise risk stratification.

### 4.3. Clinical implications and future directions

The findings of this study have direct clinical relevance. They suggest that clinical decision-making for GDM should be guided by a combined assessment of TIR and HOMA-IR. Identifying patients with the “low TIR-high HOMA-IR” phenotype is critical, as this subgroup may warrant earlier and more intensive interventions, such as stricter dietary guidance, more frequent monitoring, or a lower threshold for considering insulin therapy. Future research should focus on developing and validating personalized management protocols for this high-risk subgroup and investigating whether these findings are generalizable to GDM populations requiring pharmacological therapy.

### 4.4. Study limitations

We fully acknowledge several important limitations of this study, which also provide direction for future research:

1. Limited generalizability: To purely observe the relationship between TIR and HOMA-IR, we excluded patients requiring insulin or other glucose-lowering medications. While this ensured internal validity, it means our findings are primarily applicable to diet-controlled GDM populations, limiting their generalizability to the broader spectrum of GDM severity requiring medication.
2. Sample size and statistical power: Although the total sample size was adequate, subdivision into 4 groups resulted in limited sample size per subgroup. This reduced the statistical power to detect subtle yet clinically meaningful differences between groups (increasing the risk of Type II errors), as potentially observed with the cesarean delivery rate.
3. Inherent biases of retrospective design: As a retrospective study, our analysis is susceptible to residual confounding. While we adjusted for known confounders in our statistical models, the influence of unmeasured factors cannot be entirely ruled out.
4. Definition of HOMA-IR cutoff: The HOMA-IR cutoff value of 2.0 used for group stratification was selected for exploratory analysis based on our cohort data, as a universally accepted threshold for pregnancy is lacking. This cutoff requires validation and standardization in future large-scale, prospective studies.
5. Data presentation errors: We candidly acknowledge that the initial version of the manuscript contained errors in data transcription and presentation in the results tables. We have thoroughly re-checked and corrected all data against the original dataset in response to the reviewer’s comments. We apologize for these oversights in our initial data verification process and have implemented more stringent data management procedures.^[17,18]^

### 4.5. Conclusion

In conclusion, this study demonstrates that the combined assessment of the glycemic variability metric TIR and the IR metric HOMA-IR effectively identifies a subgroup of GDM patients at the highest risk for adverse neonatal outcomes. This integrated assessment strategy could facilitate more precise risk stratification and management in GDM. Future research should validate these findings in larger, prospective cohorts that include pharmacologically treated patients and explore pathways for translating this strategy into targeted clinical interventions.

## Acknowledgments

This study was approved by the Ethics Committee of the Gansu Province Maternity and Child-Care Hospital (No. (2022)GSFY-51). Research involving human participants was performed in accordance with the Declaration of Helsinki.

## Author contributions

**Conceptualization:** Mali Chen, Jing Yang, Yan Dong.

**Data curation:** Mali Chen, Jun Sun, Jing Yang, Jianfen Wang, Chengzhao Shu.

**Formal analysis:** Jing Yang, Jianfen Wang, Chengzhao Shu, Xiaoli Chai.

**Funding acquisition:** Yan Dong.

**Investigation:** Jun Sun, Jing Yang, Jianfen Wang, Chengzhao Shu, Xiaoli Chai.

**Methodology:** Mali Chen, Jianfen Wang, Lili Zhang.

**Resources:** Mali Chen, Jing Yang, Jianfen Wang, Chengzhao Shu.

**Software:** Mali Chen, Jianfen Wang, Chengzhao Shu.

**Supervision:** Lili Zhang, Yan Dong.

**Validation:** Mali Chen.

**Writing – original draft:** Mali Chen.

**Writing – review & editing:** Lili Zhang, Yan Dong.
